# Prevalence and determinants of hypertension among pastoralists in Monduli District, Arusha region in Tanzania: a cross-sectional study

**DOI:** 10.1186/s13690-020-00485-0

**Published:** 2020-10-14

**Authors:** Ahmed Gharib Khamis, Mbazi Senkoro, Akwilina Wendelin Mwanri, Katharina Kreppel, Sayoki Godfrey Mfinanga, Bassirou Bonfoh, Gideon Kwesigabo

**Affiliations:** 1 Department of Epidemiology and Biostatistics, Muhimbili University of Health and Allied Sciences, Dar-es-Salaam, Tanzania; 2 National Institute for Medical Research, Muhimbili Research Centre, Dar-es-Salaam, Tanzania; 3 Department of Food Technology, Nutrition and Consumer Sciences, Sokoine University of Agriculture, Morogoro, Chuo Kikuu Tanzania; 4 School of Life Sciences and Bio-Engineering, Nelson Mandela African Institution of Science and Technology, Arusha, Tanzania; 5 Department of Environmental Health and Ecological Sciences, Ifakara Health Institute, Dar-es-salaam, Tanzania; 6 Centre Suisse de Recherches Scientifiques en Côte d’Ivoire, Abidjan, Côte d’Ivoire

**Keywords:** Hypertension, Blood pressure, Diet, Physical activity, Body mass index, Pastoralist, Tanzania

## Abstract

**Background:**

Hypertension is among the growing non-communicable diseases (NCDs) in developing countries and the leading cause of death worldwide. Pastoral areas have been identified to be at a higher risk of diseases due to challenges in their daily food production, livelihoods or mobility. Unfortunately, the prevalence of hypertension and the risk factors particularly affecting rural and pastoral populations are not fully understood, making intervention efforts challenging. The aim of this study was to determine the prevalence of hypertension and identify the risk factors among adults living in Monduli district in Tanzania. The findings will be useful for the provision of tailored interventions focused on community-specific nutritional and behavioral practices.

**Methods:**

We conducted a community based cross-sectional study involving a sample of 510 adults aged above 18 years selected using a multistage cluster sampling in the Monduli district of Arusha region, Tanzania. Data were collected by using interviewer-administered questionnaires containing socio-demographic, physical activity, smoking and alcohol consumption. Anthropometry, systolic (SBP) and diastolic blood pressure (DBP) levels were measured. A one-day 24 h diet recall was conducted to evaluate the dietary habits of all participants. Both linear and logistic regression analysis were used to identify the independent predictors for hypertension and blood pressure levels.

**Results:**

The prevalence of hypertension in this study was 25.7% (*n =* 131, 95% CI; 22.1–29.7). The odds of hypertension increased with being male (AOR = 1.75, 95%CI, 1.06–2.88), belonging to the older age group of 30–39 year olds (AOR = 3.3, 95%CI, 1.76–6.38), 40–59 year olds (AOR = 3.34, 95%CI, 1.75–6.37) and ≥ 60 year olds (AOR = 4.2, 95%CI, 2.02–8.87), being overweight or obese (AOR = 3.37, 95%CI, 1.18–9.62), have more hours spent sedentary (AOR = 3.19, 95%CI, 1.61–6.32), and consumption of fatty foods (AOR = 2.23, 95%CI, 1.27–3.93). The odds for hypertension was significantly reduced among participants who reported higher income (AOR = 0.47, 95% CI, 0.25–0.91), high level of physical activity (AOR = 0.55, 95%CI, 0.31–0.96) and those reported to consume fruit (AOR = 0.37, 95% CI, 0.18–0.77). Consumption of cereals was negatively associated with levels of SBP (β = − 17.4, 95% CI, − 23.8; − 11.0) and DBP (β = − 6.6, 95% CI, − 11.5,-1.79).

**Conclusion:**

About one in every four adults living in pastoral communities have been found to have hypertension in this study. Our findings suggest that older age, obesity or overweight, low physical activity, low income, and consumption of fatty foods increase the risk of hypertension among study population. Their diet was dominated by cereals with moderate intake of meat and milk and low fruits. There is a need to promote physical activities and consumption of fruits in the study population in order to fight against hypertension. Further research should be done to confirm the associations.

## Background

Hypertension is a well-known condition causing many non-communicable diseases (NCDs) such as cardiovascular diseases, stroke, and chronic kidney disease [1–3] leading to premature mortality if not detected and treated [4]. Hypertension is therefore an important public health issue worldwide [3]. It has been estimated that the number of adults with hypertension has increased over the years from 594 million in 1975 to 1.13 billion in 2015 [5]. Globally, about one in every four men and one in every five women have hypertension [5]. The prevalence of hypertension varies substantially across regions and countries, with estimated adult prevalence in 2015 ranging from 20% in the high-income Asia Pacific region to 33% in Central and Eastern Europe, and from 11% in the high income Asia Pacific region to 28% in sub-Saharan Africa [6]. The prevalence of hypertension have been found to decline substantially in high income countries for the past few decades, and is now increasing in low- and middle-income countries [6]. In Sub-Saharan Africa (SSA) alone, more than 125 million people are expected to have hypertension by 2025 [7]. Tanzania has a rapidly increasing rate of hypertension than many other SSA countries [8–10]. A previous national representative survey conducted in Tanzania found that 26% of the adults aged 25 to 64 years have hypertension [11]. This may result in high morbidity and mortality from potentially preventable complications such as stroke and heart attack. Lifestyle, economic and socio-demographic transition of rural and urban populations have played a big role in the current rise of NCDs like hypertension in Tanzania [12]. Unfortunately, the prevalence of hypertension and the risk factors particularly affecting rural and pastoral populations are not fully understood, making intervention efforts challenging.

There are several factors that put people at risk of developing hypertension that be either modifiable or non-modifiable. Gender, age, heredity and race are among the risk factors that cannot be modified. Modifiable risk factors include lifestyle related factors such as diet, obesity, low physical activity, excessive alcohol consumption and smoking. They can also be pathologic like diabetes mellitus and hyperlipidemia [1, 4]. There have been well-established associations between the above mentioned risk factors on hypertension in different populations in various settings [13–17], but whether and how such associations exist among pastoral communities is not well studied, especially in Tanzania.

Pastoral areas has been identified to be at higher risk of food insecurity, malnutrition and various diseases due to challenges in their daily food production, livelihoods or mobility. Their diet is mainly based on milk, cereals and other animal-sourced foods, resulting in a higher risk to hypertension owing to the potentially high intake in saturated fats and salt [18–21]. Meat products are rarely consumed, where animals are slaughtered for specific occasions or social obligations [18], making their diet low in protein with higher calories. In addition, climate change, land conflict and expansion of cities dilutes the traditional pastoralism and promotes sedentarism [22], which have been previously shown to affect the health status of women and children in pastoral areas [23]. Studies conducted in pastoral communities in Tanzania and other African countries show significant higher rates of negative health consequences such as under-nutrition and certain infectious diseases [24–26]. However, there is limited and inconsistent studies conducted on the magnitude and risk factors of non-communicable diseases (NCDs) among pastoral communities in Tanzania [27–30]. This indicated that investigating the magnitude of hypertension and identifying its risk factors within this context is an important step in the design of appropriate strategies to mitigate the problem.

Assessing the magnitude and risk factors of hypertension has important public health implications in Tanzania and other developing countries, where the prevalence is still high and the lifestyle of both rural and urban population is changing rapidly [12]. It is therefore imperative that data on hypertension are determined so that stakeholders can design tailored interventions focused on community-specific nutritional and behavioral practices aimed at effectively addressing hypertension and associated NCDs. This study aimed to determine the prevalence of hypertension and assess the risk factors among adult living in pastoral communities in Monduli district in Tanzania.

## Methods

### Study design and setting

We conducted a community based cross-sectional study that enrolled adults aged ≥ 18 years old from the Monduli district of Arusha region in Tanzania, from November 2019 to February 2020. Tanzania is a country in East Africa, and is divided into 31 regions, one being Arusha region situated in the northern part of Tanzania. In Tanzania, almost 70% of the population live in rural areas, and most of them involved in the farming activities such as livestock keeping. Arusha is known to be a region representing the majority of livestock keepers in Tanzania. Monduli district is one of the seven districts of the Arusha Region. This district was purposely selected because it is known to be the homeland for pastoralist communities whose livelihood depends on livestock keeping [31]. The major ethnic group inhabiting Monduli district is the “Maasai” people (97%), and the majority are semi-nomadic pastoralists [26]. The study participants were permanent residents from five villages namely; Emaeret, Enguik, Mto wa mbu, Monduli juu and Esilalei. The district covers an area of about 6993 km^2^, and has an estimated population of 123,153. The remaining few ethnic groups like “Waarusha” live mostly in the town areas of the district. Apart from livestock keeping, people engage in cultivation of major crops and including maize, beans, paddy, wheat/barley, banana and coffee. Their livelihood depends highly on natural resources. The data were collected from November, where she short rains started, and the long rains usually comes between March and May [26]. Study participants were recruited from their traditional households (bomas), where the interview and measurements was done. We used interviewer-administered questionnaires to obtain information about socio-demographic characteristics, physical activity and 24-h diet recall. The questionnaires were modified from the WHO STEPS tool for NCDs, and were pre-tested before used in this study. With the help of three research assistants (RAs), data were directly captured in mobile phones and tablets using Open Data Kit software (ODK collect version 1.10).

### Sample size and sampling

We calculated the sample size by assuming a hypertension prevalence of 22% in a similar setting [27], 5% precision at 95% confidence level, 5% non-response rate, and design effect of 1.5, which as a result gives a total sample size of 474. Finally, we managed to conduct interviews of 510 participants. Probability sampling, in particular multistage cluster sampling, utilizing the administrative structure as the sampling frame was used to select participants. Village is the lowest administrative unit in Tanzania. We obtained the list of all villages from the Monduli district office. Five (5) of the 62 listed villages were randomly selected and their respective village chairperson provided a household sampling frame. The numbers of households were selected randomly proportionate to the village sizes. All adults in the household meeting the inclusion criteria were invited to participate in the study. Community health workers from the villages helped in locating the selected households.

### Inclusion and exclusion criteria

Participants were eligible to participate if they were 18 years or older, were permanent residents of the selected villages for at least 6 months [15], able to provide informed consent, and willing to follow the study procedures. This included answering questions on diet, physical activity, anthropometric and blood pressure measurements. Exclusion criteria included being pregnant or having any chronic condition resulting in diet restriction.

### Anthropometric measurement

Anthropometric measurements including weight and height were taken using a digital weighing machine (SECA 813, USA), and a non-stretchable measuring tape (Limoko tape, CHINA) respectively. Body weight was measured to the nearest 0.1 kg precision. Height was measured using a measuring tap fixed on a height board to the nearest 0.1 cm precision. Participants were asked to remove shoes and heavy clothes before weight and height were measured. All anthropometric measurements were repeated at least two times for accuracy. Body mass index (BMI) was calculated as weight (kilograms) divided by squared height (meters) and then categorized as per World health organization (WHO) standards of < 18.5 kg/m^2^ as underweight, 18.5–25 kg/m^2^ as normal, 25–30 kg/m^2^ as overweight, and above 30 kg/m^2^ as obesity. Waist circumference was measured at the thinnest point of the abdomen at the end of a normal expiration. Hip circumference was measured at the maximum circumference over the buttock horizontally. Both waist and hip circumferences were measured using a measuring tape with 1 mm accuracy. Waist-hip ratio (WHR) was calculated by dividing the waist by the hip circumference. Abdominal obesity was defined based on the WHO cut-offs, whereby measurement of WHR ≥0.90 in males and WHR ≥0.85 in females indicates higher risk for cardiovascular diseases [32].

### Blood pressure measurement

Blood pressure was measured at least three times by trained health professionals. The participants rested for few minutes before their blood pressure was measured. All three measurements were performed in a sitting position for at least five minutes apart. The average of last two readings were used for analysis. Blood pressure was measured using a validated digital automatic blood pressure machine (ProLogic PL100). Measurement was done on the upper left arm with an appropriate cuff size. Both systolic blood pressure (SBP) and diastolic blood pressure (DBP) were measured together with pulse rate (PR). According to the WHO and American Heart Association —hypertensive person was identified if SBP ≥ 140 mmHg and/or DBP ≥ 90 mmHg or self-reported use of anti-hypertensive medications [33].

### Physical activity measurement

Physical activity was assessed by using Global Physical Activity Questionnaire (GPAQ version 2) endorsed by the WHO for its STEP wise Approach to NCDs risk factors surveys [34]. This was done by asking participants to recall and estimate the amount of time they spend doing different types of activity including working (digging, lifting heavy objects), walking (herding animals), sports and recreational, and sedentary activities. The physical activity level was measured by metabolic equivalents (MET) in minutes per week in the domain of occupation, transportation, and recreational activity, and it was divided into three groups as: low physical activity (≤599 MET-min/week), moderate physical activity (600–2999 MET-min/week), and high physical activity (≥3000 MET-min/week) based on GPAC classification [27]. Sedentary time (hours/minutes) was calculated by asking each participant to report the number of hours spent at leisure in a day and was divided into four categories: No sedentary hour, 1–2 h/day, 3–5 h/day, ≥ 6 h/day.

### Dietary assessment

To collect the dietary intake information, we asked all participants to recall their previous 24-h foods and beverages consumed including foods eaten outside the home. A dietary diversity score (DDS) was constructed according to the Food and Agriculture Organization (FAO) guidelines for measuring individual dietary diversity [35]. According to FAO protocol, reported food items were categorized into 12 food groups which are (i) cereals; (ii) legumes; (iii) roots and tubers; (iv) vegetables (v) fruits (vi) milk and dairy products; (vi) eggs; (vii) meat; (viii) fats; (ix) fish; (xi) sweets/sugary and (xii) beverages and condiments. Then, an aggregated DDS was created by summing the number of food groups consumed (possible range from zero indicating the participant ate none of the food groups; to 12 indicating that he/she ate all the food groups). To understand if previous 24-h consumption was typical or not, we asked them to respond to a question “Was yesterday a normal day where you ate special foods, or ate more, or less than usual? As a proxy of food insecurity, we asked them to report their usual number of meals per day.

### Other explanatory variables

Information on age, gender, education, income, diabetes history, lifestyle (smoking, alcohol), history of diagnosis and use of medication for hypertension was self-reported by a participant. Age was categorized into four common groups: 18–29 years (young), 30–39 years (middle-aged adults), 40–59 (mature adult) and 60 years or above (old adults). We divided educational attainment into five groups following the school system in Tanzania. The groups were no education, primary education, secondary education (O-level), advanced secondary education (A- level) and college or University. Monthly income of participants was defined as total earnings for the subjects in the previous month, and was divided into; less than 50,000 (22 USD), between 50,000-200,000, and above 200,000, as expressed in Tanzania shillings (TSH). We adopted questions from the WHO STEPS survey tool to ask the participants if they have been previously diagnosed with diabetes and/or hypertension, or taking diabetes medication at the time of the study. Smokers were defined as subjects who had responded “Yes” to the question “Have you ever smoked/used tobacco product in your entire life? Alcohol consumption was categorized into: Never, occasional (one to three drinks per week), and frequent (more than three drinks per week).

### Statistical analysis

The data were cleaned and analysed in Stata (Stata 13/SE, StataCorp, College Station, TX), and Statistical Package for Social Science (SPSS) version 23. Age (years), Weight (kg), height (cm) and other anthropometric variables were presented as numerical variables. Other characteristics like physical activity, educational attainment and hypertension were defined as categorical variables, and the prevalence of hypertension was estimated. The categorical variables were compared between male and female and using Chi-square test. Numerical data were compared between gender by independent *t*-test or Mann-Whitney test. Linear and logistic regression analysis were conducted to identify independent predictors of blood pressure levels and hypertension. Stepwise selection procedures using backward elimination method in multivariate logistic regression model were used to identify independent predictors of hypertension and control for confounders. The main outcome of interest was a dichotomous dependent variable of hypertension (1 = hypertension; 0 = no hypertension). Based on previous literature on hypertension and from the bivariate results (if *p <* 0.2), the following variables were included into the multivariate logistic regression model; (1) age, (2) gender,(3) Overweight or obesity (BMI) (4) abdominal obesity, (5) physical activity, (6) sedentary time, (7) smoking, (8) alcohol consumption, (9) consumption of cereals, (10) consumption of fruits, (11) consumption of milk, (12) consumption of meat, (13) consumption of fatty foods, (14) education,(15) number of meals, (16) income, (17) and history of diabetes. We check for multicollinearity using estimates of the variance inflation factor (VIF) of the variables included in the model, and VIF value below 10 was considered acceptable. All crude (OR) and adjusted odds ratios (AOR) of hypertension with 95% confidence intervals (CIs) were two-sided and considered significant if *p* ≤ 0.05 in the multivariate model.

## Results

### Characteristics of the participants

A total of 510 adults aged 18 years and above participated in this study. Their median (IQR) age was 36(52–25) years, of whom 241(47.2%) were males. The majority (37.3%) were aged between 18 and 29 years, followed by 40–59 years (27.3%). Primary school education was reached by 52.2%, and very few (6.5%) were educated up to university or college level. Male participants were significantly taller than females, however, female participants had higher BMI, larger waist and hip circumferences, and lower physical activity level compared to males (*p* < 0.001) (Table 1). There was significant difference between male and female participants with regard to mean systolic (SBP), but not diastolic blood pressure (DBP), or mean pulse rate. Moreover, about 12.2% were smokers, and 25.3% were frequent alcohol users. About 44% of participants have low physical activity and the majority of them were females. Generally, 47.7% of males were found to have high physical activity level, while only 32.3% of females engaged in high physical activity. Details about their socio-demographic characteristics are shown in Table 1.

**Table 1:** The characteristics of the study participants in Monduli District in Tanzania, 2020 (*n* = 510)

Variables	Mean ± SD or ***n*** (%)			***P-***value
	Overall	Women	Men	
	***n*** = 510	***n*** = 269	***n*** = 241	
Weight (kg)	67.1 ± 14.6	68.5 ± 16.9	65.5 ± 11.7	0.081
Height (cm)	162.9 ± 9	157.9 ± 7.3	168.1 ± 7.2	< 0.001
Waist circumference (cm)	83.8 ± 16.8	85.3 ± 19.1	81.8 ± 13.3	< 0.001
Hips circumference (cm)	97.6 ± 13.2	102.1 ± 16.9	92.6 ± 10.3	< 0.001
Waist hips ratio (WHR)	82.4 ± 14.6	85.3 ± 15.3	79.6 ± 13.2	0.027
Systolic blood pressure (mmHg)	127.8 ± 18.3	126 ± 18.5	129.6 ± 18.1	0.022
Diastolic blood pressure (mmHg)	78.1 ± 13.6	78.2 ± 12.8	78.1 ± 14.5	0.845
Pulse rate (beats/min)	78.9 ± 20.1	79.9 ± 22.1	77.8 ± 17.6	0.243
Number of meals	2.7 ± 0.5	2.7 ± 0.5	2.7 ± 0.5	0.351
Dietary diversity score (DDS)	4.4 ± 2.1	4.2 ± 2.2	4.4 ± 2.1	0.161
**Age (years)**				
Median (IQR)	36(52–25)	39(57–25)	34(50–25)	
18–29	190(37.3)	94(34.9)	96(39.8)	0.003
30–39	92(18)	45(16.7)	47(19.5)	
40–59	139(27.2)	67(24.9)	72(29.8)	
Above 60	89(17.5)	63(23.4)	26(10.8)	
**Educational attainment**				
No education	110(21.6)	63(23.4)	47(19.5)	0.393
Primary education	226(52.2)	136(50.6)	130(53.9)	
O level secondary	20(3.9)	8(2.9)	12(5)	
A level secondary	81(15.9)	47(17.5)	34(14.1)	
College/University	33(6.5)	15(5.6)	18(7.5)	
**Marital status**				
Single	80(15.6)	34(12.6)	46(19.1)	< 0.001
Married/cohabiting	353(69.2)	172(63.9)	181(75.1)	
Divorced	32(6.7)	23(8.5)	9(3.7)	
Widow	45(8.8)	40(14.8)	5(2.1)	
**Physical activity (PA)**				
Low	225(44.1)	135(49.8)	90(36.9)	0.002
Moderate	86(16.8)	48(17.8)	38(15.4)	
High	199(39.1)	86(32.3)	113(47.7)	
**Body mass index (BMI)**				
Underweight	42(8.2)	29(10.8)	13(5.4)	< 0.001
Normal	239(46.8)	80(29.7)	159(65.9)	
Overweight	126(24.7)	63(23.4)	63(26.1)	
Obesity	103(20.2)	97(36.1)	6(2.5)	
**Abdominal obesity (WHR)**				
Normal	317(62.2)	140(52.1)	177(73.4)	< 0.001
Obesity	193(37.8)	129(47.9)	64(26.6)	
**Monthly income (Tsh)**				
No income	140(27.4)	86(31.9)	54(22.4)	
< 50,000	145(28.4)	95(35.3)	50(20.8)	< 0.001
50,000-200,000	100(19.6)	36(13.4)	64(26.6)	
> 200,000	125(24.5)	52(19.3)	73(30.3)	
**Smoking**				
Ever experience	62(12.2)	12(4.5)	50(20.8)	< 0.001
Never experience	448(87.8)	257(95.5)	191(79.2)	
**Alcohol consumption**				
Never	354(69.4)	197(73.2)	157(65.2)	0.048
Occassional	27(5.3)	9(3.3)	18(7.5)	
Frequent	129(25.3)	63(23.4)	66(27.4)	

### Prevalence of hypertension

As shown in Table 2, there were 131 (25.7% CI; 22.1–29.7) people with hypertension in this study. The gender specific prevalence of hypertension was significantly higher among males 27.5% (95% CI; 22.2–33.5) than females 24.1% (95% CI; 19.4–29.6) (*p* < 0.001). For both genders, the prevalence of hypertension increased with the age from the youngest age group of 18–29 years (13.5% male; female 12.7%) to the older age group of 40–59 years (42.2% male; female 26.8%) and oldest of 60 years (42.3% male; female 42.8%).

**Table 2:** Prevalence of hypertension stratified by age and gender of the pastoralists in Monduli District, Tanzania, 2020 (*n* = 510)

Age (years)	Male		Female		Overall	
	***n***	% (95% CI)	***n***	% (95% CI)	***n***	% (95% CI)
18–29	13	13.5 (7.9–22)	12	12.7 (7.3–21.2)	25	13.1 (9.0–18.8)
30–39	12	25.5 (14.9–40)	8	17.7 (9.0–31.9)	20	21.7 (14.4–31.4)
40–59	30	42.2 (31.2–54.1)	18	26.8 (17.5–38.8)	48	34.7 (27.2–43.1)
≥ 60	11	42.3 (31.2–54.1)	27	42.8 (31.1–55.4)	38	42.6 (32.7–53.2)
**Total**	**66**	**27.5 (22.2–33.5)**	**65**	**24.1 (19.4–29.6)**	**131**	**25.7 (22.1–29.7)**

*CI* confidence interval.

### Dietary habits

**Fig. 1:**
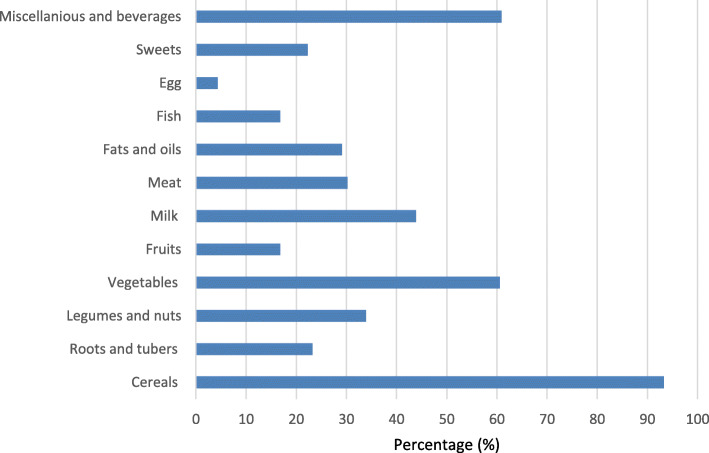
Percentage of consumption of foods from different food groups in pastoral communities in Monduli District, Tanzania, 2020

Figure 1 shows the percentage of individuals who reported to consume specific food groups in the previous day before the interview. It was found that nearly all 93.3% (*n* = 473) participants had consumed foods made of cereals such as rice, maize and maize meal. In addition, just over one third of the participants 33.9% (*n* = 173) consumed food items made from legumes such as beans. It was found that the percentage of vegetables consumption 60.6% (*n* = 309) was higher than fruits 16.8% (*n* = 86). This was followed by the consumption of fatty foods 29.1% (*n* = 148), meat 30.2% (*n* = 154) and sweets/sugary foods 22.3% (*n* = 114). Beverages such as soft drinks, tea and coffee 60.9% (*n* = 311) were mostly consumed rather than milk 43.9% (*n* = 224). It was also found that very few participants consumed eggs 4.3% (*n* = 22). As a results their mean (SD) dietary diversity score (DDS) was 4.4 (2.1) whereby, about 44.9% reported to consume less than 4 food groups out of 12. In addition, the majority (91%) of the respondents said that this previous 24-h consumption represent their typical consumption of foods. The mean (SD) number of meals reported by participants was 2.7 (0.5).

### Factors associated with hypertension

Table 3 presents the crude and adjusted odds ratio from a bivariate and multivariate logistic regression model to identify independent predictors of hypertension. This study revealed the following as the predictors of hypertension in the final model: (1) age, (2) gender, (3) overweight or obesity (BMI), (4) physical activity, (5) income, (6) abdominal obesity, (7) sedentary time,(8) fats consumption, (9) cereals consumption, (10) and fruits consumption. Variables that did not appear in the final multivariate logistic regression model are:- (1) consumption of milk, (2) consumption of meat; (3) alcohol consumption; (4) history of diabetes; (5) educational attainment; (6) number of meals, (7) smoking. In this study, the odds of hypertension was found to be higher among males than females (AOR = 1.75, 95%CI, 1.06–2.88). Likewise, the odds of hypertension was significantly higher among those aged between 30 and 39 years (AOR = 2.1, 95%CI, 1.02–4.36), 40–59 (AOR = 3.34, 95%CI, 1.75–6.37), and ≥ 60 years (AOR = 4.1, 95%CI, 1.82–8.02) compared with individuals aged 18–29 years. Compared to underweight, overweight or obese participants had more than three times the odds of being hypertensive (AOR = 3.33, 95%CI, 1.37–8.09). As expected, this study revealed that there was a significant association between physical activity level and hypertension. Participants who showed higher levels of physical activity had lower odds of hypertension compared to those with lower physical activity (AOR = 0.55, 95%CI, 0.31–0.96). Moreover, the odds of getting hypertension for those who have more than 6 h per day of sedentary time were more than three times as high compared to those who have no sedentary hours (AOR = 3.19, 95%CI, 1.61–6.32). In terms of food consumption, this study shows the increased odds of having hypertension for individual who consumed fatty foods (AOR = 2.23, 95%CI, 1.27–3.93), but the odds were reduced, albeit not significantly for those who consumed foods made of cereals (AOR = 0.42, 95%CI, 0.18–1.07). Nevertheless, the odds of having hypertension were found to be significantly reduced for those who reported to consume fruits (AOR = 0.37, 95%CI, 0.18–0.77) and those having higher income (AOR = 0.47, 95% CI, 0.25–0.91) (Table 3). To confirm whether the blood pressure level is associated with the above factors, linear regression analysis was performed. Table 4 shows that age, BMI, waist and hip circumferences, abdominal obesity (WHR), and consumption of cereals were the variables that best explaining the variations of systolic and diastolic blood pressure. In this analysis, total metabolic time (MET), income and hours spent sedentary have explained little variations in the linear regression model.

**Table 3:** Bivariate and multivariate logistic regressions for risk factors of hypertension among adults in pastoral communities in Monduli District, Tanzania, 2020 (*n* = 510)

	Hypertension (***n*** = 131)	Crude OR (***n*** = 510)	Adjusted OR (***n*** = 510)
**Variables**	***n*(%)**	**OR (95% CI)**	**AOR(95% CI)**
**Age**			
18–29	25(19.1)	Ref	Ref
30–39	20(15.3)	1.83(0.95–3.51)	2.11(1.02–4.36)*
40–59	48(36.6)	3.52(2.1–6.09)***	3.34(1.75–6.37)***
Above 60	38(29)	4.92(2.71–8.91)***	4.1(1.82–8.02)***
**Gender**			
Female	65(49.6)	Ref	Ref
Male	66(50.4)	1.2(0.8–1.77)	1.75(1.06–2.88)*
**Monthly income (Tsh)**			
No income	47(35.9)	Ref	Ref
< 50,000	32(24.4)	0.56(0.33–0.95)*	0.47(0.25–0.91)*
50,000-200,000	16(12.2)	0.37(1.9–0.71)**	0.45(0.21–1.49)
> 200,000	36(27.5)	0.81(0.48–1.36)	0.79(0.43–1.49)
**Body Mass Index (BMI)**			
Underweight	7(5.3)	Ref	Ref
Normal	42(32.1)	1.06(0.44–2.56)	1.34(0.47–3.42)
Overweight or Obese	50(38.2)	3.33(1.37–8.09)**	2.72(1.27–7.1)*
**Abdominal Obesity**			
Normal	59(45.1)	Ref	Ref
Obesity	72(54.9)	2.59(1.73–3.89)***	1.56(0.95–2.59)
**Sedentary time per day (hours)**			
No	48(36.6)	Ref	Ref
1 to 2	32(24.4)	0.87(0.52–1.46)	1.36(0.63–2.16)
3 to 5	13(9.9)	0.36(0.18–0.71)**	0.52(0.24–1.13)
≥ 6	38(29)	2.1(1.2–3.5)**	3.19(1.61–6.32)**
**Physical activity category (PA)**			
Low	72(54.9)	Ref	Ref
Moderately	24(18.3)	0.823(0.47–1.42)	0.87(0.44–1.72)
High	35(26.7)	0.45(0.28–0.72)**	0.55(0.31–0.96)*
**Consumption of cereals**			
No	18(13.7)	Ref	Ref
Yes	113(86.3)	0.24(0.11–0.5)***	0.42(0.18–1.07)
**Consumption of fatty foods**			
No	87(66.4)	Ref	Ref
Yes	44(33.6)	1.33(0.86–2.03)	2.23(1.275–3.93)**
**Consumption of fruits**			
No	117(89.3)	Ref	Ref
Yes	14(10.7)	0.51(0.27–0.94)*	0.37(0.18–0.77)**

*P < .05, **P < .01, ***P < 0.001; *CI* confidence interval, *OR* Odd ratio. Only variables significantly associated with hypertension in the multivariate logistic regression were shown

**Table 4:** Linear regression of factors associated with levels of systolic and diastolic blood pressure among pastoral communities in Monduli district in Tanzania, 2020 (*n* = 510)

Variables	Dependent variable (SBP) (mmHg)		Dependent variable (DBP) (mmHg)	
	***β*** (95% CI)	***R***^**2**^ (%)^**a**^	***β*** (95% CI)	***R***^**2**^ (%)^**a**^
Age (years)	4.06(2.7;5.42)***	6.3	3.3(2.28;4.3)***	7.5
Gender (Males)	4.01(0.84;7.18)*	1.2	−2.3(−2.6;2.1)	< 0.1
Body mass index (kg/m2)	0.82(0.57;1.08)***	7.5	0.62(0.43;0.81)***	7.6
Waist circumference (cm)	0.28(0.18;0.38)***	6.1	0.2(0.14;0.29)***	6.1
Hip circumference (cm)	0.16(0.06;0.25)**	2.2	0.15(0.07;0.22)***	3.2
Abdominal obesity (WHR)	31.1(15.9;46.3)***	3.3	15.5(3.86;27.13)**	1.4
Metabolic time (MET/week)	−0.19(−0.37;-0.01)*	0.8	−0.06(− 0.19;0.07)	0.1
Sedentary time (hours/day)	0.75(0.22;1.28)**	1.5	0.4(0.01;0.79)*	0.8
Income (Tsh)	−0.2(−1.6;1.18)	< 0.1	0.1(−0.9;1.15)	< 0.1
Number of meals	−0.39(− 0.41;3.24)	< 0.1	− 1.97(−4.6;0.74)	0.4
Consumption of fruits ^b^	−1.93(−6.2; 2.3)	0.1	− 1.3(− 4.5;1.8)	0.1
Consumption of cereals ^b^	−17.4(−23.8;-11.0)***	5.3	−6.6(− 11.5;-1.79)**	1.4
Consumption of fatty foods ^b^	1.6(−1.85;5.16)	0.1	2.16(−4.5;4.77)	0.5
Dietary diversity score	−0.05(− 0.78;-0.67)	< 0.1	0.03(− 0.51;0.57)	< 0.1

*P < .05, **P < .01, ***P < 0.001; *β =* Unadjusted Regression coefficient*; CI* confidence interval, *a* Coefficient of determination, *b* food groups coded as binary 0 = if not consumed and 1 = if consumed, *SBP* Mean systolic blood pressure, *DBP* Mean diastolic blood pressure, *mmHg* millimeter of mercury

## Discussion

This study aimed to determine the prevalence of hypertension and provides a description of the associated factors among adults living in pastoral communities in Tanzania. This may have important implications for interventions tackling cardiovascular diseases like hypertension. About one in every four (26%) adults aged 18 years old have been found to have hypertension in this study. This is similar to the Tanzanian national prevalence estimated previously [11]. This prevalence is also comparable with several studies conducted in Tanzania [10, 36] and some sub-Sahara African countries [17, 37]. This is not the first study to reveal a prevalence of hypertension among pastoralists in Tanzania. According to Petrucka et al. [27], the prevalence of hypertension among pastoralists residing in Simanjiro district was 21.3%, while in Morogoro district was 29.3% [30], and that reported by Diarz et al. [21] in Ngorongoro district was 9.8%. These differences in prevalence may be due to the variations in lifestyle risk factors in these communities.

This study shows that male gender, higher BMI, lower income, low physical activity, sedentary behavior, and consumption of fatty foods with less fruits were associated with higher odds of hypertension. This therefore revealed a closely related pattern of risk factors as described in several other studies of non-pastoralists populations [16, 17]. It has been reported that people living in urban areas are exposed to more sedentary activities and consumption of fatty foods than rural or pastoral populations [10, 38]. It is more likely that pastoral communities are undergoing a lifestyle transformation to a more urban way of lifestyle.

In the current study, we found a significant associations between consumption patterns of specific food groups and hypertension. The association between dietary pattern and hypertension is well established [37]. High fat and salt, low in fruit and vegetables, and with meat intakes was sometimes found to be associated with hypertension [13, 17, 27]. Our study found that consumption of fatty foods can increase the risk for hypertension. This study however did not find the association between meat and milk consumption with hypertension, as opposed to previous studies [13, 21]. Lawson et al. [18] found that pastoralists were rarely consuming animal products like meat and eggs, except on traditional occasions and during rituals. Furthermore, Njelekela et al. [38] reported that participants from the pastoral communities were found to consume high amounts of milk and have been found to have low blood pressure levels. A review of recent literature shows that consumption of milk and dairy products have a beneficial effect on blood pressure [39]. Based on this study, consumption of meat and milk may not be among the potential dietary risk factors of hypertension among pastoralists living in this community. Moreover, it has been stated previously that pastoralists are changing their livelihood and consumption patterns [19, 40]; hence these changes of specific foods like meat and milk will be likely accompanied with changes in their traditional dietary pattern. Despite the fact that consumption of fruits was low in this study, we found that it reduce the odds of getting hypertension. The protective effect of fruits could be due to high micronutrients present or good diet diversification for those who consume fruits, which improve their overall health status. Fruits and vegetable consumption was reported to be protective in other studies as well [27]. Therefore, these findings illuminated the importance of dietary interventions for prevention of hypertension among the study populations.

We also found that overweight or obese participants have higher odds of having hypertension. Our findings are generally consistent with previous body of evidence [27, 28, 41, 42]. In addition, epidemiological studies are suggesting that obesity is not just a factor associated with higher levels of blood pressure, but may causally be associated with it [43, 44]. Thus, overweight or obesity plays a critical role in the development of cardiovascular diseases like hypertension among these communities. Expectedly, we found that the likelihood of hypertension increased among older and male participants. The age group of 60 years and above was the most affected with hypertension in this study. The main reason for this may be due to natural physiological processes in which, blood vessels lose elasticity with advancing age [28]. Age is a common non-modifiable risk factor for many non-communicable diseases (NCDs) which is not affected by environmental factors. Therefore, it is possible that population ageing will increase the prevalence of hypertension in this community. Despite being more physically active and being less obese than females, male subjects showed a higher likelihood of hypertension than female. As reported by Everett et al. [45], lower levels of awareness and healthcare seeking behavior were found for males and identified as key risk factors for developing hypertension.

This study clearly indicates that moderate to high physical activity levels have a protective effect against hypertension and high blood pressure levels. The WHO has recommended that an adult should achieve at least 600 metabolic equivalents per week, which is equivalent to 150 min of moderate physical activity, or 75 min of high physical activity. Overall, the majority of participants in this study falls below this recommendation, and the majority were females (49.8%) than males (36.9%). Normally, among pastoralists, males walk long distances daily to herd cattle while the females and elderly stay home and perform light work [27]. The low physical activity in this study may be also due to marginalization, vulnerability to drought and land conflicts, which are some of the known causes forcing pastoralist to become more sedentary [18]. In addition, our findings show that participants with no income are more likely to have hypertension compared with those from high income. On the other hand, the likelihood of hypertension increases by more than twice for those having more sedentary time. Sedentary activities are highly associated with overweight or obesity, both of which increase the risk of many other NCDs like cardiovascular diseases and type 2 diabetes [46]. Therefore, this finding confirms the widely recognized benefits doing regular physical activity and restriction of sedentary activities for improved health.

On the other hand, smoking and alcohol consumption are among the leading causes of morbidity and mortality from NCDs around the world [47]. In this study, we did not find an association between smoking and hypertension. It should be noted that, the prevalence of smoking in this study was lower (12.2%) compared to the national estimated prevalence of 14.1% [11]. Likewise, we did not find an association between alcohol consumption with hypertension in this study, potentially due to small amount consumed, which was not investigated in the current study. This finding is consistent with a study conducted by Mosha et al. [16].

There are few limitations to our study. The cross-sectional nature of this study prevents the identification of the causal pathways underlying the reported associations. We also did not collect the quantity of foods consumed like amount of salt and cholesterol intake, which might have profound effects on hypertension. The dietary information was collected over a one-day recall period, which may not be a true reflection of the long-term dietary patterns. However, this does not discredit the validity and reliability of 24-h dietary diversity in measuring the dietary habits of the diverse populations [48]. This study did not take into account other medical conditions like the use of drugs, oral contraceptives or other hormonal treatments that may also confound the associations. Furthermore, the use of proxy-reported questionnaires, which are subject to errors like recall bias may result in a certain degree of mis-reporting or over-reporting. This study has some notable strengths. We conducted this study in pastoral communities and collected information on the behavioral risk factors like diet, physical activity, smoking and alcohol consumption. The results from this study can be generalized to several locations in the country with similar ethnic groups like Maasai in order to design NCDs prevention programs.

## Conclusion

In conclusion, about one in every four adults living in pastoral communities were found to have hypertension in this study. The prevalence is much higher among males than females. Older age, obesity or overweight, low physical activity level, and consumption of fatty foods increase the likelihood of having hypertension among pastoralists. Fruit consumption is associated with lower odds of having hypertension. The increase in sedentary activity will make people in this community more vulnerable to hypertension. There was a moderate consumption of meat and milk which was not found to be among the independent predictors of hypertension and blood pressure levels. Therefore, results presented in this study emphasize the role of interventions that target diet and physical activity for the prevention of hypertension and high blood pressure levels in this community. Further research is needed to determine habitual dietary intake more precise to confirm the associations.

## Data Availability

The data used to support the findings of this study are available from authors upon special request.

## Abbreviations

BMI: Body mass index
BP: Blood pressure
DBP: Diastolic blood pressure
DDS: Dietary diversity score
HTN: Hypertension
MET: Metabolic equivalent time
NCDs: Non-communicable diseases
PA: Physical activity
SBP: Systolic blood pressure
WHR: Waist to hip ratio
WHO: World Health Organization
